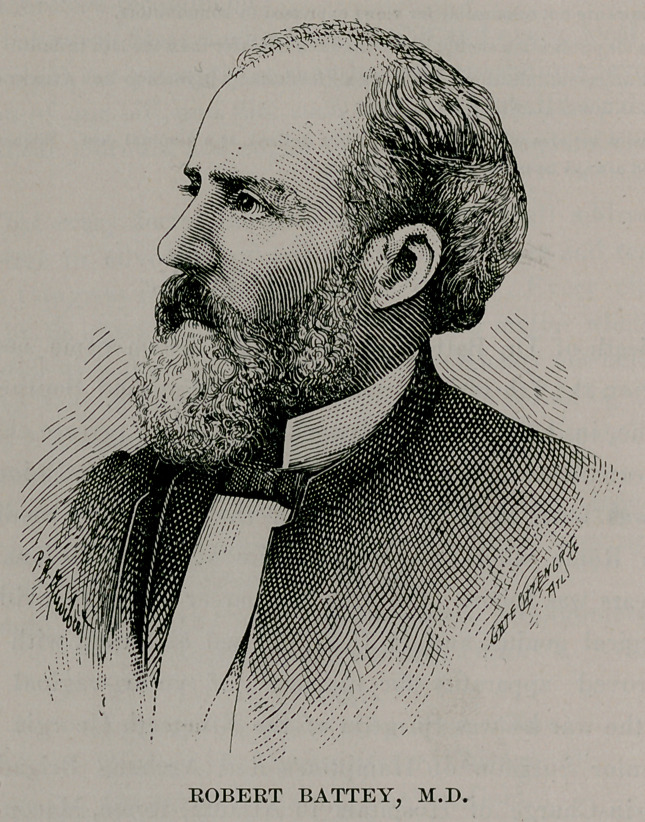# Dr. Robert Battey

**Published:** 1895-12

**Authors:** 


### *EDITORIAL.*

---

The office of the Business Manager of THE JOURNAL is Nos. 311 and 312 Fitten building.

The editors are not responsible for views expressed by contributors.

Articles for publication should reach this office not later than the 15th instant.

Address all communications, and make all remittances payable to THE ATLANTA MEDICAL AND SURGICAL JOURNAL, Atlanta, Ga.

Reprints of articles will be furnished, when desired, at a nominal cost. Requests for the same should always be made *on the manuscript.*

---

### DR. ROBERT BATTEY.

The death of Dr. Battey, of Rome, Ga., which came not unexpectedly on the 7th ultimo, removes a distinguished Southern surgeon, who, in his professional work and in his private character, was an ornament and an honor to the medical profession. Dr. Battey was born in 1828, and began the practice of medicine in 1857 in Rome, where nearly his entire active professional life of thirty years was spent. Early in his career he gave evidence of high surgical genius, and, in 1859, devised and used with success an improved apparatus for the cure of vesico-vaginal fistula. During the war he was Surgeon of the Nineteeth Georgia Volunteers, Senior Surgeon of Hampton's and Archer's Brigades, and Surgeon-in-Charge of Hospitals in Atlanta, Rome, Macon, Vineville, and Lauderdale, Miss. In 1869, Dr. Battey was the first to put into execution the suggestion of Dr. Willard Parker by performing perineal section for a case of chronic cystitis. In 1874 he performed with success vaginal ovariotomy, which was the third instance of this operation. Dr. Battey was the first Georgia surgeon to perform ovariotomy. This was done in 1867, for the removal of a thirty-pound dermoid cyst. But it was the operation of August 17, 1872, that made Dr. Battey famous. Ovaries were not removed so ruthlessly then as now, and when it was proposed by Dr. Battey in such general terms to perform the operation in order to "establish at once the change of life for the effectual remedy of certain otherwise incurable maladies," the proposition

ROBERT BATTEY, M.D.

was regarded as a species of surgical vandalism. The profession was indignant. Even Marion Sims was "astounded at the audacity of the operation." However, professional sentiment gradually changed, and the operation was admitted, even by those who had vigorously opposed it at first, to be based upon sound physiological doctrine. At this juncture there arose two other claimants to the credit of priority, but medical history has accorded to Dr. Battey the credit of introducing the operation and of the moral courage involved in it.

Dr. Battey was universally highly esteemed, both in his professional relations and in his private life. His distinguished skill and ability gained for him professional honors, at home and abroad, of which any one could be justly proud. Some of these we have spoken of in our October issue. While living in Atlanta, in 1874, he was active in the reorganization of the ATLANTA MEDICAL AND SURGICAL JOURNAL, and for many years this JOURNAL contained frequent able articles from his pen.

The death of such men as Dr. Battey is a loss to any community and to the profession of which he was an honorable and honored member. It is doubtful whether we will see his like again. Dr. Battey was pre-eminently a representative of that class known as a "gentleman of the old school," to whom the principle of *noblesse oblige* was something more than an empty motto. He died at the age of sixty-seven, leaving behind him the record of a life devoted to good works.

## Figures and Tables

**Figure f1:**